# Future directions in prevention and treatment of children obesity and eating disorders

**DOI:** 10.1186/1824-7288-41-S2-A72

**Published:** 2015-09-30

**Authors:** Rita Tanas, Guido Caggese, Simonetta Marucci

**Affiliations:** 1 UO Pediatria, Azienda Ospedaliero Universitaria, Ferrara, Italy; 2 Formazione Professionale, Azienda Ospedaliero Universitaria, Ferrara, Italy; 3 Centro per il trattamento dei Disturbi del Comportamento Alimentare, Todi, Perugia, Italy

## 

The childhood obesity epidemic has promoted many healthcare programs focused on nutritional education in order to teach correct nutrition and to promote early diagnosis and care of overweight/obese children including offering them a correct food supply.

For almost 20 years, however, scientific literature has pointed out that eating disorders (ED), which typically onset during adolescence, are increasing, and that dieting (restrictive behaviors, adopted today by many adults and adolescents, mostly managed without the support of a professional format, to match the ideal of thinness proposed by the media and being or feeling overweight are likely to be the most avoidable facilitating factors [1-4].

Body stigma, shared by those with obesity and ED [5] and based on the attribution of a strong personal responsibility in the onset of these diseases, has been shown to increase stress, blood pressure, cortisol, oxidative stress, C-reactive protein and worse glycemic control, while decreasing motivation for physical activity (PA) encouraging a sedentary lifestyle. Stigma also increases psychosocial issues, such as depression, body-image distortion, loss of oral control and affects motivation and effectiveness of treatments.

Primary care staff are increasingly asked to promote parents awareness on their children being overweight although this has been recognized as a trigger for the onset of an ED [6], but at the same time they are not provided with adequate means to run a truly effective therapeutic program (strategies to promote change, such as the patient-centered motivational interview) and the suffering related to family and professional weight-derision.

Children are then pushed to see themselves as fat, to dislike themselves and go on a diet, focusing dangerously on food, body and weight and with the result of being bullied in many life contexts such as home, school, medical services. All this leads to dysfunctional eating patterns, loss of control and increased BMI. It has been reported that obesity is the strongest predictive factor to the onset of an ED [7]. The pervasive and universal body and weight-stigma enhances restrictive behavior and creates a vicious circle that leads to a real ED epidemic among young adults [8]. It's time to think about obesity prevention and treatment in a different way (Tables 1 and 2), getting to know EDs [8,9] and using tools that do not favor them, protecting children against weight-stigma, especially ones coming from parents, educators and health workers [10].

**Table 1:** Multiple strategies for a comprehensive systemic-oriented approach to childhood obesity and ED [11-14].

Actions	Setting
Regulating the commerce of energy-dense nutrition-poor foods, cosmetics and cosmetic surgery for minors to their health needs	Governments, Schools

Controling sophisticated marketing strategies of unhealthy food, sedentary activities and weight loss products to which children are often exposed	Governments, Media

Restricting the use of very underweight fashion models (BMI<18) and digitally-altered photographs of models	Governments, Media

Protecting people from weight discrimination and bullism, both in workplaces and clinical settings	Governments, Media

Educating people eating healthy food and physical activities and marketing	Families, School educators

Offering healthy food and promote physical activities during school-time and after-school	Schools, Community

Training on prevention and early diagnosis of eating disorders and weight-related diseases	Schools, Sports coach, Healthcare

Promoting attention and prevention to professional anti-fat bias in school and clinical practice	Schools, Sports coach, Healthcare

Handling with weight-stigmatized or bullied children	Schools, Healthcare

Training on patient-centered motivational counselling	Healthcare

Empowering children to cope with media-based and culturally inappropriate messages and promoting acceptance of the diversity of beauty and attractiveness, enhancing inner qualities versus appearance and expression of discomfort.	Schools

**Table 2:** Themes for Professional Training about Childhood Obesity for Primary Care (PC) and Multiprofessional Teams (Pediatricians, hospital, nutritionists, psychologists).

Number	Items	Healthcare
**1**	Epidemiology and Etiology update	PC

**2**	Diagnosis criteria: use of BMI z score according to the WHO growth charts	PC

**3**	Principles of Motivational Interviewing and its application in Primary Care	PC and Team

**4**	Brief Motivational Counseling with families and adolescents for the communication of the diagnosis and the support to the motivation to behaviors’ change	PC and Team

**5**	Developing awareness on empathy and derision towards people with obesity.	PC and Team

**6**	Handling with weight and body-stigma promoted by families, school educators and health professionals	PC and Team

**7**	Proactive assessment of the outcome of care and its communication to families	PC and Team
